# Supplementary material for: Evaluation of reporting quality of randomized controlled trials in patients with COVID-19 using the CONSORT statement
Source: PLoS One. 2021 Sep 23;16(9):e0257093. doi: 10.1371/journal.pone.0257093 (PMC8460279; doi:10.1371/journal.pone.0257093)
Supplement: S1 File — (DOCX) [file pone.0257093.s001.docx]

**PubMed** December 1, 2020

Search Strategy:

| **#** | **Searches** | **Results** |
| --- | --- | --- |
| 1 | "novel coronavirus"[Title/Abstract] OR "novel coronavirus 2019"[Title/Abstract] OR "2019-nCoV"[Title/Abstract] OR "COVID-19"[Title/Abstract] OR "coronavirus disease"[Title/Abstract] OR "Wuhan coronavirus"[Title/Abstract] OR "Wuhan pneumonia"[Title/Abstract] OR "SARS-CoV-2"[Title/Abstract] OR "SARS 2"[Title/Abstract] OR "severe acute respiratory syndrome coronavirus 2"[Title/Abstract] OR "coronavirus disease 2019 virus"[Title/Abstract] | 69971 |
| 2 | "Randomized Controlled Trials as Topic"[Mesh] | 141203 |
| 3 | "randomized controlled trial"[Publication Type] OR "controlled clinical trial"[Publication Type] OR "randomized"[Title/Abstract] OR "placebo"[Title/Abstract] OR "randomly"[Title/Abstract] OR "trial"[Title] | 1198429 |
| 4 | #2 **OR** #3 | 1262585 |
| 5 | #1 **AND** #4 | **1480** |

**Embase** December 1, 2020

Search Strategy:

| **#** | **Searches** | **Results** |
| --- | --- | --- |
| 1 | 'randomized controlled trial'/exp | 628522 |
| 2 | 'randomization'/exp | 88788 |
| 3 | 'controlled clinical trial'/exp | 800876 |
| 4 | randomized:ti,ab,kw OR randmised:ti,ab,kw OR randomly:ti,ab,kw OR random*:ti,ab,kw | 1604215 |
| 5 | #1 **OR** #2 **OR** #3 **OR** #4 | 1886402 |
| 6 | 'novel coronavirus':ti,ab,kw OR 'novel coronavirus 2019':ti,ab,kw OR '2019-ncov':ti,ab,kw OR 'covid-19':ti,ab,kw OR 'coronavirus disease':ti,ab,kw OR 'wuhan coronavirus':ti,ab,kw OR 'wuhan pneumonia':ti,ab,kw OR 'sars-cov-2':ti,ab,kw OR 'sars 2':ti,ab,kw OR 'severe acute respiratory syndrome coronavirus 2':ti,ab,kw OR 'coronavirus disease 2019 virus':ti,ab,kw | 66789 |
| 7 | #5 **AND** #6 | 1768 |

**Cochrane Library** December 1, 2020

Search Strategy

| **#** | **Searches** | **Results** |
| --- | --- | --- |
| 1 | MeSH descriptor: [Randomized Controlled Trials as Topic] explode all trees | 14539 |
| 2 | (randomized controlled trial):pt OR (controlled clinical trial):pt OR (randomized):ti,ab,kw OR (placebo):ti,ab,kw OR (randomly):ti,ab,kw OR (trial) :ti,ab,kw | 1326375 |
| 3 | #1 **OR** #2 | 1326377 |
| 4 | (novel coronavirus):ti,ab,kw OR (novel coronavirus 2019):ti,ab,kw OR (2019 nCoV):ti,ab,kw OR (COVID-19):ti,ab,kw OR (coronavirus disease):ti,ab,kw OR (Wuhan coronavirus):ti,ab,kw OR (Wuhan pneumonia):ti,ab,kw OR (SARS-CoV-2):ti,ab,kw OR (SARS 2):ti,ab,kw OR (severe acute respiratory syndrome coronavirus 2):ti,ab,kw OR (coronavirus disease 2019 virus):ti,ab,kw | 3401 |
| 5 | #3 **AND** #4 | **2714** |

**Web of Science** December 1, 2020

Science Citation Index Expanded (SCI-EXPANDED) – from 2019 to now

Search Strategy

| **#** | **Searches** | **Results** |
| --- | --- | --- |
| 1 | TS=("novel coronavirus" OR "novel coronavirus 2019" OR  "2019-nCoV" OR "COVID-19" OR "coronavirus disease" OR "Wuhan coronavirus" OR "Wuhan pneumonia" OR "SARS-CoV-2" OR "SARS 2" OR "severe acute respiratory syndrome coronavirus" OR "coronavirus disease 2019 virus") | 48565 |
| 2 | TS=("randomized controlled trial" OR "controlled clinical trial" OR randomized OR placebo OR randomly OR trial) | 265833 |
| 3 | #1 **AND** #2 | **2738** |
