# Supplementary material for: Evaluation of reporting quality of randomized controlled trials in patients with COVID-19 using the CONSORT statement
Source: PLoS One. 2021 Sep 23;16(9):e0257093. doi: 10.1371/journal.pone.0257093 (PMC8460279; doi:10.1371/journal.pone.0257093)
Supplement: Attachment — Submitted filename: PONE-D-21-13616_reviewer.pdf [file pone.0257093.s002.pdf]

PLOS ONE

Reporting quality of randomised controlled trials regarding patients or suspected patients with COVID-19

--Manuscript Draft--

**Manuscript Number:**	PONE-D-21-13616
**Article Type:**	Research Article
**Full Title:**	Reporting quality of randomised controlled trials regarding patients or suspected patients with COVID-19
**Short Title:**	Reporting quality of randomised controlled trials regarding patients or suspected patients with COVID-19
**Corresponding Author:**	Juxia Zhang<br>Gansu Provincial Hospital<br>Lanzhou, CHINA
**Keywords:**	COVID-19; patients or suspected patients; randomised controlled trials, reporting quality
**Abstract:**	Objective: To evaluate the reporting quality of randomised controlled trials (RCTs) regarding patients or suspected patients with COVID-19.<br>Methods: The following databases were PubMed, Embase, Web of Science and The Cochrane Library databases to collect RCTs related to patients or suspected patients with COVID-19. The retrieval time was from the inception to December 1, 2020. The CONSORT 2010 checklist was used to evaluate the reporting quality of these RCTs. Results: A total of 53 RCTs were included, The CONSORT checklist showed that the average reporting rate of all items was 52.68%, among which the average reporting rate of the method section was 31.51%. Mean overall adherence across all studies was 13.02±3.546, 95%CI (12.06–13.97) and the scores ranged from 7 to 22. Overall reporting quality is suboptimal. The multivariate linear regression analysis showed the reporting scores were influenced significantly by types of intervention (P=0.009), the first author's countries are China (P=0.001) and America (P=0.025), journal impact factor (P=0.003), and journal with endorsement of CONSORT statement (P=0.015).<br>Conclusion: Although many RCTs of regarding patients or suspected patients with COVID-19 have been published in different journals, the reporting quality of these studies were of concern. The overall reporting quality of RCTs is suboptimal, thus diminishing their potential usefulness,and it is not conducive to the rapid and effective control of COVID-19. Therefore, more journals should accept the CONSORT statement, and researchers should also attach great importance to the reporting standardization of RCTs.
**Order of Authors:**	Yuhuan Yin
	Yiyin Zhang
	Xiaoli Zhang
	Jianying Ye
	Juxia Zhang
**Additional Information:**	
**Question**	**Response**
**Financial Disclosure**<br><br>Enter a financial disclosure statement that describes the sources of funding for the work included in this submission. Review the submission guidelines for detailed requirements. View published research	The author(s) received no specific funding for this work.

Powered by Editorial Manager® and ProduXion Manager® from Aries Systems Corporation

articles from *PLOS ONE* for specific examples.<br><br>This statement is required for submission and **will appear in the published article** if the submission is accepted. Please make sure it is accurate.<br><br>**Unfunded studies**<br>Enter: *The author(s) received no specific funding for this work.*<br><br>**Funded studies**<br>Enter a statement with the following details:<br>• Initials of the authors who received each award<br>• Grant numbers awarded to each author<br>• The full name of each funder<br>• URL of each funder website<br>• Did the sponsors or funders play any role in the study design, data collection and analysis, decision to publish, or preparation of the manuscript?<br>• **NO** - Include this sentence at the end of your statement: *The funders had no role in study design, data collection and analysis, decision to publish, or preparation of the manuscript.*<br>• **YES** - Specify the role(s) played.<br><br>* typeset	
**Competing Interests**<br><br>Use the instructions below to enter a competing interest statement for this submission. On behalf of all authors, disclose any competing interests that could be perceived to bias this work—acknowledging all financial support and any other relevant financial or non-financial competing interests.<br><br>This statement is required for submission and **will appear in the published article** if the submission is accepted. Please make sure it is accurate and that any funding sources listed in your Funding Information later in the submission form are also declared in your Financial Disclosure statement.	The authors have declared that no competing interests exist.

View published research articles from *PLOS ONE* for specific examples.<br><br>**NO authors have competing interests**<br><br>Enter: *The authors have declared that no competing interests exist.*<br><br>**Authors with competing interests**<br><br>Enter competing interest details beginning with this statement:<br><br>*I have read the journal's policy and the authors of this manuscript have the following competing interests: [insert competing interests here]*<br><br>* typeset	
**Ethics Statement**<br><br>Enter an ethics statement for this submission. This statement is required if the study involved:<br><br>• Human participants<br>• Human specimens or tissue<br>• Vertebrate animals or cephalopods<br>• Vertebrate embryos or tissues<br>• Field research<br><br>Write "N/A" if the submission does not require an ethics statement.<br><br>General guidance is provided below. Consult the submission guidelines for detailed instructions. **Make sure that all information entered here is included in the Methods section of the manuscript.**	N/A

**Format for specific study types**<br><br>**Human Subject Research (involving human participants and/or tissue)**<br>• Give the name of the institutional review board or ethics committee that approved the study<br>• Include the approval number and/or a statement indicating approval of this research<br>• Indicate the form of consent obtained (written/oral) or the reason that consent was not obtained (e.g. the data were analyzed anonymously)<br><br>**Animal Research (involving vertebrate animals, embryos or tissues)**<br>• Provide the name of the Institutional Animal Care and Use Committee (IACUC) or other relevant ethics board that reviewed the study protocol, and indicate whether they approved this research or granted a formal waiver of ethical approval<br>• Include an approval number if one was obtained<br>• If the study involved *non-human primates*, add *additional details* about animal welfare and steps taken to ameliorate suffering<br>• If anesthesia, euthanasia, or any kind of animal sacrifice is part of the study, include briefly which substances and/or methods were applied<br><br>**Field Research**<br><br>Include the following details if this study involves the collection of plant, animal, or other materials from a natural setting:<br>• Field permit number<br>• Name of the institution or relevant body that granted permission	
**Data Availability**<br><br>Authors are required to make all data underlying the findings described fully available, without restriction, and from the time of publication. PLOS allows rare exceptions to address legal and ethical concerns. See the PLOS Data Policy and FAQ for detailed information.	Yes - all data are fully available without restriction

A Data Availability Statement describing where the data can be found is required at submission. Your answers to this question constitute the Data Availability Statement and **will be published in the article**, if accepted.<br><br>**Important:** Stating 'data available on request from the author' is not sufficient. If your data are only available upon request, select 'No' for the first question and explain your exceptional situation in the text box.<br><br>Do the authors confirm that all data underlying the findings described in their manuscript are fully available without restriction?	
**Describe where the data may be found in full sentences. If you are copying our sample text, replace any instances of XXX with the appropriate details.**<br><br>• If the data are **held or will be held in a public repository**, include URLs, accession numbers or DOIs. If this information will only be available after acceptance, indicate this by ticking the box below. For example: *All XXX files are available from the XXX database (accession number(s) XXX, XXX.).*<br>• If the data are all contained **within the manuscript and/or Supporting Information files**, enter the following: *All relevant data are within the manuscript and its Supporting Information files.*<br>• If neither of these applies but you are able to provide **details of access elsewhere**, with or without limitations, please do so. For example:<br><br>*Data cannot be shared publicly because of [XXX]. Data are available from the XXX Institutional Data Access / Ethics Committee (contact via XXX) for researchers who meet the criteria for access to confidential data.*<br><br>*The data underlying the results presented in the study are available from (include the name of the third party*	All relevant data are within the manuscript and its Supporting Information files.

and contact information or URL).<br>• This text is appropriate if the data are owned by a third party and authors do not have permission to share the data.<br><br>* typeset	
Additional data availability information:	

Manuscript Click here to access/download;Manuscript;Manuscript.docx

Reporting quality of randomised controlled trials regarding patients or suspected patients with COVID-19

Yuhuan Yin[1], Yiyin Zhang[1], Xiaoli Zhang[1], Jianying Ye[1], Juxia Zhang*,[2]

[1] School of Nursing, Gansu University of Chinese Medicine, Lanzhou, China, 730000

[2] Clinical Educational Department, Gansu Provincial Hospital, Lanzhou, Gansu, China, 730000

* **Corresponding author:**

Juxia Zhang, Professor

Email: 1425331412@qq.com

Clinical Educational Department,Gansu Provincial Hospital, Lanzhou, Gansu, China

Abstract

Objective: To evaluate the reporting quality of randomised controlled trials (RCTs) regarding patients or suspected patients with COVID-19.

Methods: The following databases were PubMed, Embase, Web of Science and The Cochrane Library databases to collect RCTs related to patients or suspected patients with COVID-19. The retrieval time was from the inception to December 1, 2020. The CONSORT 2010 checklist was used to evaluate the reporting quality of these RCTs.

Results: A total of 53 RCTs were included, The CONSORT checklist showed that the average reporting rate of all items was 52.68%, among which the average reporting rate of the method section was 31.51%. Mean overall adherence across all studies was 13.02±3.546, 95%CI (12.06–13.97) and the scores ranged from 7 to 22. Overall reporting quality is suboptimal. The multivariate linear regression analysis showed the reporting scores were influenced significantly by types of intervention (P=0.009), the first author's countries are China (P=0.001) and America (P=0.025), journal impact factor (P=0.003), and journal with endorsement of CONSORT statement (P=0.015).

Conclusion: Although many RCTs of regarding patients or suspected patients with COVID-19 have been published in different journals, the reporting quality of these studies were of concern. The overall reporting quality of RCTs is suboptimal, thus diminishing their potential usefulness,and it is not conducive to the rapid and effective control of COVID-19. Therefore, more journals should accept the CONSORT statement, and researchers should also attach great importance to the reporting standardization of RCTs.

Key words: COVID-19; patients or suspected patients; randomised controlled trials, reporting quality

Introduction

In December 2019, a β-coronavirus was discovered in Wuhan, China. The World Health Organization (WHO) has named the new disease as Coronavirus disease 2019 (COVID-19), and Coronavirus Study Group (CSG) of the International Committee has named it as SARS-CoV-2 [1]. Globally, as of 17 March 2021, there have been 120,164,106 confirmed cases of COVID-19, including 2,660,422 deaths [2]. COVID-19 is a new acute respiratory infectious disease caused by SARS-CoV-2 infection and it is highly contagious and spreads quickly. It mainly infringes the respiratory system, and at the same time, it may infringe various organs of the whole body and present corresponding symptoms, with fever, dry cough and fatigue as the main clinical manifestations. Some patients have diminished or lost sense of smell and taste as the first symptoms, in addition, a small number of patients have symptoms such as nasal congestion, runny nose, sore throat, conjunctivitis, myalgia and diarrhea. The average incubation period is five to six days, but it can be as long as 14 days [3]. COVID-19 primarily spreads through the respiratory tract, droplets, respiratory secretions and direct contact for a low infective dose [4]. COVID-19 is mainly treated by Western medicine, and can also be treated by integrated Chinese and Western medicine [3]. Currently, a large number of RCTs regarding patients or suspected patients with COVID-19 have been published.

Randomised controlled trials (RCTs) are the gold standard to compare the effectiveness of different interventions [5]. By which health care professionals make decisions about the efficacy and effectiveness of interventions. Evaluating the validity of trial is dependent on the quality and transparency of study report [6]. Thus, if the design is not scientific, the implementation process is not rigorous, the result analysis is not appropriate and the report is not standard, then the real effect of the intervention will be biased [7]. This can adversely influence meta-analysis findings and clinical practice recommendations [8].

Due to poor reporting of randomized controlled trials, the CONSORT (reporting standards of randomized controlled trials) statement was developed in 1996 [9], and revised in 2001 [10] and 2010 [11] to maximize the reporting quality of randomized controlled trials and provide the detail and transparency for readers. The updated CONSORT statement includes a checklist of 25 items that list the key items that should be included in the RCT, with titles and abstracts, methods, results, discussions and other information. However, since many of the 25 items were subdivided into two subitems, the list actually consists of 37 items [11]. It is currently known to be endorsed by over 600 biomedical journals and endorsed by several prominent editorial organizations including the International Committee of Medical Journal Editors (ICMJE) and the World Association of Medical Editors (WAME) [12]. After update of CONSORT statement, the overall reporting quality of RCTs has improved. however, the quality of many RCTs are still not optimistic at present, and there are some defects in each link of the research report [13,14]. The results of RCTs related to

ophthalmic surgery performed by researchers in the United Kingdom showed The mean CONSORT score was 8.9 (range 3.0-14.7) and the reporting quality was low [15]. A study of RCTs from Thailand regarding herbal Interventions showed that adherence to the recommended CONSORT checklist items for reporting of RCTs ranged from 0% to 97.18% [16], this means that some RCTs have a CONSORT rate of 0%. However, to our knowledge, no study has specifically evaluated the reporting quality of RCTs in COVID-19.

In summary, the completeness of the report is important for the clinician to understand the full value of the study. The primary objective of our study was to assess the compliance of the reporting of RCTs in patients or suspected patients with COVID-19 to the 2010 CONSORT checklist and analyze possible related causes, and it provides theoretical basis for subsequent related studies and meta-analysis, and provides suggestions for RCTs of higher reporting quality.

Methods

Ethical review

Ethical approval was not necessary for this study as the study did not involve patient, and the included RCTs can be traced from database.

Search strategy

The following databases were PubMed, Embase, Web of Science and The Cochrane Library databases to collect RCTs related to patients or suspected patients with COVID-19. The retrieval time was from the inception to December 1, 2020. The search terms included:COVID-19, novel coronavirus 2019, novel coronavirus,

randomized controlled trial, RCT, etc. The search was conducted by two investigators and the detailed strategy was shown in S1 Appendix.

Study selection

For included studies must meet the following criteria: study design: clinical randomized controlled trial. Subjects: confirmed or suspected patients with COVID-19. The control group was set up, and the intervention measures of the experimental group were not limited and full text can be obtained.

There were no limitations in techniques or styles of interventions. The retrieved literature titles were imported into the Endnote X9. Titles and abstracts of initially retrieved studies were screened by 2 reviewers independently. The full texts of eligible reviews and uncertain reviews were obtained and examined to determine whether they met the inclusion criteria. Any disagreements were resolved by discussion.

Data extraction

Two authors independently extracted the general characteristics and reporting data of the included studies into Excel. The general characteristics include country, number of authors, impact factor of journals, sample size, age and types of participants, journals' endorsement of the CONSORT statement. The CONSORT statement was chosen as a tool to assess reporting quality for this study [11]. We assess the compliance of each report with 25 items in CONSORT statement. If item wasdescribed in the report, each checklist item and subitem was answered with “yes” or “no”. According to the above items, the coincidence rate of each item of included 53 studies was counted one by

one. Total scores of items 1–25 were then calculated, with a point for each item being granted if all sub-sections were scored as yes or half a point if one out of the two sub-sections were reported[5]. Items 3b, 6b, and 14b are non-essential. If relevant, the article will be graded according to the above guidelines if this information is not provided. If the article does not apply to this entry, no points will be deducted [7].

Statistical analysis

Descriptive statistics was performed to describe general characteristics included studies and the overall reporting quality and the reporting rate of each checklist item/subitem. Statistical analysis was carried out using SPSS version 22, We analyzed the possible associations of the reporting quality of included 53 studies and independent predictors including country, number of authors, impact factor of journals, sample size, age and types of participants, whether journals requires a citation of CONSORT statement using multiple linear regression analyses, $p < 0.05$ was considered statistically significant. Where appropriate, 95% confidence intervals (CI) were also calculated.

Results

Search results

Initially, 8700 articles were obtained. Excluding duplicates, 6,922 studies was remained. After screening the titles and abstracts, 198 potentially eligible reviews were identified. Subsequently full text of each article was retrieved with a total of 53 RCTs that were confirmed for further assessment. Figure 1 outlines the search detail via the PRISMA flow diagram.

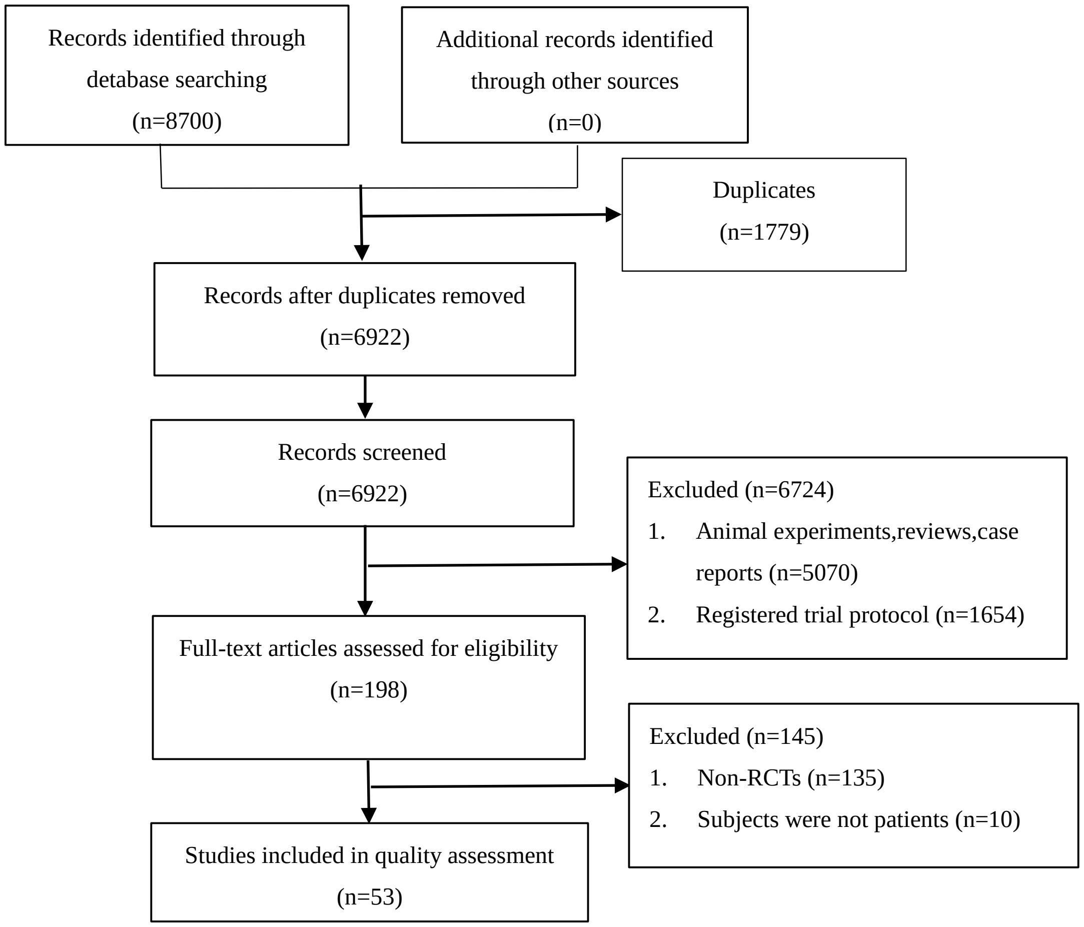

Figure 1. Literature screening process and results

Characteristics of included studies

Among the included 53 RCTs, China published the most articles, accounted for 49.06%. The number of authors is mainly between 21 and 30. 50 studies (94.34%) used drug interventions. 29 studies (54.72%) had an impact factor of journals less than 5, but 12 studies (22.64%) also had a journal impact factor of more than 30. 25

studies (47.17%) did not explicitly require authors to cite the CONSORT statement. In addition, all the included studies were published in 2020 and multicenter study. As showed in Table 1.

Table 1. Characteristics of included studies.

Category	Item	Number of articles n (%)	CONSORT scores ($\bar{x}$±SD)
Country of first author	China	26 (49.06)	13.50±4.032
	America	8 (15.09)	13.56±3.478
	Iran	6 (11.32)	10.67±2.066
	Other countries	13 (24.53)	12.81±2.876
Number of authors	1～10	9 (16.98)	11.33±3.162
	11～20	13 (24.53)	10.96±2.436
	21～30	18 (33.96)	13.53±3.070
	＞30	13 (24.53)	15.54±3.816
Sample size	＜100	25 (47.17)	11.18±2.610
	100～300	17 (32.08)	14.41±3.308
	＞300	11 (20.75)	14.14±3.233
Participants	suspected cases	11 (20.75)	12.73±2.563
	confirmed cases	42 (79.25)	13.08±3.774
Age of participants (years)	18～60	41 (77.36)	13.39±3.658
	＞60	2 (3.77)	11.00±1.414
	others	10 (18.87)	11.75±2.870

Interventions	psychology	3 (5.66)	8.33±1.528
	drugs	50 (94.34)	13.30±3.439
Journal Impact Factor for 2019	＜5	29 (54.72)	10.79±2.254
	5～10	6 (11.32)	12.50±1.708
	10～30(Don‘t contain 10)	5 (9.43)	15.20±1.891
	＞30	13 (24.53)	16.73±3.510
Endorsement of CONSORT	No	25 (47.17)	10.90±2.363
	Yes	28 (52.83)	14.91±3.375

Others: refers to the age of unlimit, including patients of any age.

All the included studies were published in 2020.

The evaluation result of the CONSORT checklist

The CONSORT checklist showed that the average reporting rate of all items was 52.68%, among which the average reporting rate of the method section was 31.51%. Items with the reporting rate of more than 80% were abstract, background, eligibility criteria for participants, outcomes, statistical methods, participant flow chart, baseline data, limitations, generalisability and funding. Except for non-essential items, the remaining items with the reporting rate of less than 20% were trial design, sample size, allocation concealment, implementation, blinding, ancillary analyses , protocol. Only 7 studies (13.21%) reported methods for masking concealment and 9 studies (16.98%) reported the implementation of the blinding. In addition, the reporting rate of random sequences in the method section was also low. 26 studies (49.06%)

mentioned the method of random sequence generation.while the other 27 studies (50.94%) only mentioned randomness and did not explicitly report the specific method. Table 2 outlines the reporting frequency of each checklist item.

Table 2. The evaluation result of the CONSORT checklist

Section /Topic	Item	Item No	Checklist item	Number of articles/n (%)
Title and abstract		1a	Identifification as a randomised trial in the title	41 (77.36)
		1b	Structured summary of trial design, methods, results,and conclusions	45 (84.91)
Introduction	Background	2a	Scientifific background and explanation of rationale	53 (100.00)
	objectives	2b	Specifific objectives or hypotheses	39 (73.58)
Methods	Trial design	3a	Description of trial design (such as parallel, factorial)including allocation ratio	5 (9.43)
		3b	Important changes to methods after trial commencement (such as eligibility criteria), with reasons	0 (0.00)
	Participants	4a	Eligibility criteria for participants	47 (88.68)
		4b	Settings and locations where the data were collected	41 (77.36)
	Interventions	5	The interventions for each group with suffificient details to allow replication, including how and when they were actually administered	53 (100.00)
	Outcomes	6a	Completely defifined pre-specifified primary and secondary	50 (94.34)

		outcome measures, including how and when they were assessed	
	6b	Any changes to trial outcomes after the trial commenced, with reasons	0 (0.00)
Sample size	7a	How sample size was determined	5 (9.43)
	7b	When applicable, explanation of any interim analyses and stopping guidelines	3 (5.66)
Randomisation:	8a	Method used to generate the random allocation sequence	26 (49.06)
Sequence generation	8b	Type of randomisation; details of any restriction(such as blocking and block size)	26 (49.06)
Allocation concealment mechanism	9	Mechanism used to implement the random allocation sequence(such as sequentially numbered containers),describing any steps taken to conceal the sequence until interventions were 0assigned	7 (13.21)
Implementation	10	Who generated the random allocation sequence, who enrolled participants, and who assigned participants to interventions	0 (0.00)
Blinding	11a	If done, who was blinded after assignment to interventions (for example, participants, care providers, those assessing outcomes) and how	9 (16.98)
	11b	If relevant, description of the similarity of interventions	9 (16.98)
Statistical methods	12a	Statistical methods used to compare groups for primary and secondary outcomes	53 (100.00)
	12b	Methods for additional analyses, such as subgroup analyses and	4 (7.55)

			adjusted analyses	
Results	Participant flow(a diagram is strongly recommended)	13a	For each group, the numbers of participants who were randomly assigned, received intended treatment, and were analysed for the primary outcome	43 (81.83)
		13b	For each group, losses and exclusions after randomisation,together with reasons	0 (0.00)
	Recruitment	14a	Dates defifining the periods of recruitment and follow-up	32 (60.37)
		14b	Why the trial ended or was stopped	3 (5.66)
	Baseline data	15	A table showing baseline demographic and clinical characteristics for each group	53 (100.00)
	Numbers analysed	16	For each group, number of participants (denominator) included in each analysis and whether the analysis was by original assigned groups	35 (66.04)
	Outcomes and estimation	17a	For each primary and secondary outcome, results for each group, and the estimated effect size and its precision (such as 95% confifidence interval)	42 (79.25)
		17b	For binary outcomes, presentation of both absolute and relative effect sizes is recommended	28 (52.83)
	Ancillary analyses	18	Results of any other analyses performed, including subgroup analyses and adjusted analyses, distinguishing pre-specifified from exploratory	4 (7.55)
	Harms	19	All important harms or unintended effects in each group	41 (77.36)

Discussion	Limitations	20	Trial limitations, addressing sources of potential bias, imprecision, and, if relevant, multiplicity of analyses	51 (96.23)
	Generalisability	21	Generalisability (external validity, applicability) of the trial fifindings	51 (96.23)
	Interpretation	22	Interpretation consistent with results, balancing benefifits and harms, and considering other relevant evidence	44 (83.01)
Other information	Registration	23	Registration number and name of trial registry	38 (71.70)
	Protocol	24	Where the full trial protocol can be accessed, if available	6 (11.32)
	Funding	25	Sources of funding and other support (such as supply of drugs), role of funders	46 (86.79)

The total CONSORT scores for each study

The overall CONSORT score for each study was 13.02±3.546, 95%CI (12.06–13.97) and the scores ranged from 7 to 22. Figure 2 shows a bar chart with frequencies of total scores from all papers.

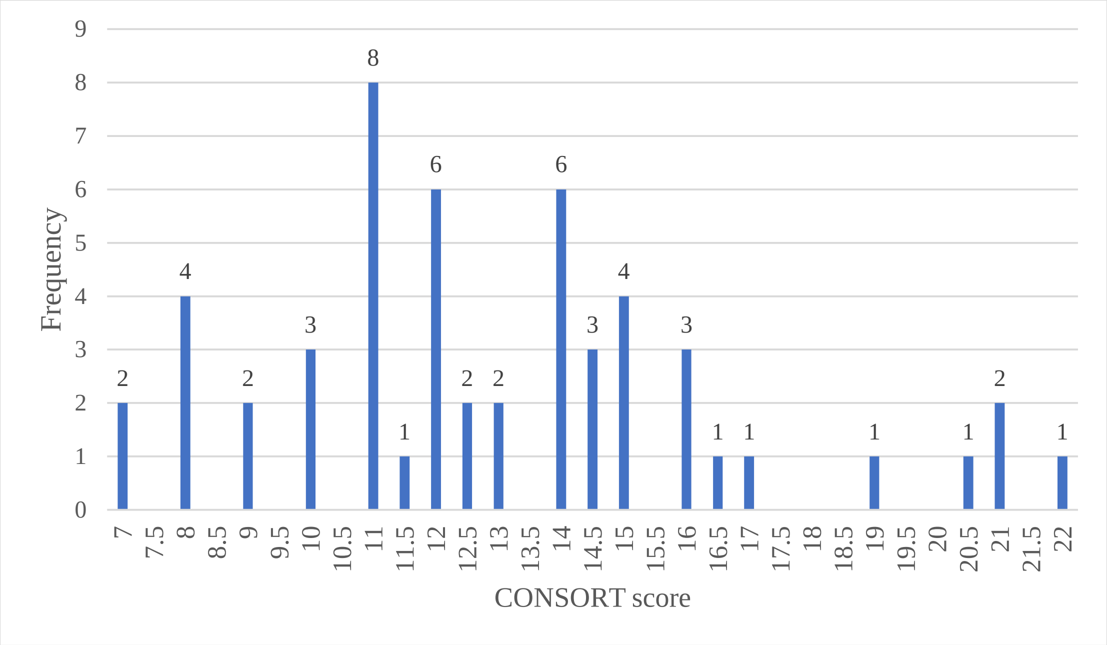

Figure 2. The frequencies of the total CONSORT scores for each study

Factors of effecting overall reporting reporting score

As shown in Table 1, The first author of the study in America has the highest adherence score of 13.56±3.478, China published the highest number of RCTs in the analyzed period and adherence score was 13.50±4.032. Studies with more than 30 authors had a total adherence score of 15.54±3.816, and drug interventions (13.30±3.439). The highest mean CONSORT score was observed in the journals with an impact factor more than 30 (16.73±3.510), while the journal with endorsement of CONSORT statement had a total adherence score of 14.91±3.375.

Table 3 displays the results of the linear regression analysis. It is showed that types of intervention (P=0.009), Journal Impact Factor for 2019 (P=0.003), and journal with endorsement of CONSORT statement (P=0.015) were associated with a significant difference in reporting quality. We used other countries as a reference, China

(P=0.001) and America (P=0.025) had the higher adherence of CONSORT checklist. No significant associations were found regarding other variables such as number of authors, sample size, etc.

Table 3. Multiple linear regression determinants of reporting quality of RCTs

	Unstandardized Coefficients		Standardized Coefficients			95% CI	
Characteristics	B	SE	Beta	t	P	Lower	Upper
Country of first author							
(1) China	0.684	0.182	0.388	3.757	0.001	0.317	1.052
(2) America	0.461	0.199	0.234	2.317	0.025	0.060	0.862
Number of authors	0.040	0.029	0.149	1.409	0.166	-0.017	0.098
Sample size	0.000	0.003	-0.019	-0.142	0.888	-0.006	0.005
Participants	0.619	0.843	0.071	0.734	0.467	-1.082	2.319
Age of participants	0.657	0.737	0.078	0.892	0.378	-0.829	2.143
Types of interventions	3.804	1.389	0.249	2.738	0.009	1.002	6.605
Journal Impact Factor for 2019	0.063	0.020	0.449	3.183	0.003	0.023	0.103
Endorsement of CONSORT	1.963	0.774	0.277	2.534	0.015	0.401	3.524

95% CI, 95% confidence interval for B; B, Spearman–Brown coefficient; SE, standard error.

Discussion

To our knowledge, this is the first study evaluating the reporting quality of RCTs regarding COVID-19. We evaluated the reporting quality of included 53 RCTs using

the CONSORT statement [11]. Our study showed that the overall reporting quality in RCTs of patients or suspected patients regarding COVID-19 is suboptimal, it is clear that the reporting quality of these RCTs needs to be improved, particularly in terms of methods section. This may be due to the urgency of epidemic prevention and control. In order to present positive results to readers within a short period of time, many scholars ignored the standardization of the report.

China published the most articles, which may be related to the outbreak of COVID-19 in China first. Therefore, compared with other countries, research institutions and medical units in China attach great importance to COVID-19 and actively carry out related RCTs. As we can see from the number of authors, 44 studies (83.02%) had more than 10 authors, mainly from 21 to 30. It shows that the focus of COVID-19 comes from society and groups as a whole, not just individuals. Because randomized controlled trials are time-consuming and costly, it is difficult for individuals to complete a high-quality RCT [17]. And the Chow study showed that the number of signed authors in RCTs reflects the degree of cooperation in scientific research, and it increases the authenticity and reliability of the results [18]. It may be difficult to recruit patients due to the urgency of the epidemic. Our results showed that 26 studies focused on a sample size of less than 100. In 2001, Sterne et al proposed for the first time that studies with small sample sizes tend to have beneficial results, which is called small sample size effect [19]. Small sample size RCTs may be associated with lower methodological quality, when the sample size was larger, the same therapeutic effect reached a statistically significant level due to the larger

sample size [20]. The small sample size effect was also verified in a large meta-epidemiological study of 735 RCTs by Dechartres et al [21]. Therefore, it is necessary to be more cautious in interpreting the RCTs results with a small sample size.

The overall reporting quality of included 53 RCTs is suboptimal. We found that none of the RCTs reported all of the CONSORT checklist items. The items with better reporting quality were mainly abstract, background, eligibility criteria for participants, outcomes, statistical methods, participant flow chart , baseline data ,limitations, generalisability and funding. Clear titles, abstracts and background enable readers to search the literature accurately and quickly in the database and help readers understand the scientific nature and necessity of the measure [22]. The standardized description of relevant information of subjects in the form of charts can enable readers to better understand the trial results, successfully repeat the trial process, and enhance the universality and application of the intervention methods[22]. Reporting the limitations of the RCTs and the reasons for potential bias and inaccuracies, as well as the reasons for multiple analysis results, can enable readers to understand the results of RCTs more objectively and comprehensively [23].

Items that drive down reporting rates were concentrated in the methodological section,such as trial design, sample size, allocation concealment, implementation, blinding, ancillary analyses and protocol. The complete description of the trial design can provide readers with accurate research ideas and enable readers to better evaluate the research results [23]. To provide a true, reliable assessment of effectiveness, reporting of random sequences, masking, and blinding are important for RCT. There

was the lack of report for descriptions of these items in our study. Amrita's study showed that the benefit of randomization is to balance the treatment groups in terms of baseline variability, known and unknown confounders, thus eliminates "confounding bias" [24]. In our study, only half of the studies described the method of random sequences, one of them was grouped by bed order, which was not truly random. Therefore, the possibility of bias is high, which makes the evidence users question the reliability of conclusion. Our study results showed that only 7 studies (13.21%) reported methods for masking concealment. 15 articles (32.08%) mentioned the blinding, but only 9 studies (16.98%) reported the implementation of the blinding. The result of a combined analysis of meta-epidemiological studies demonstrated that trials with inadequate or unclear allocation concealment overestimated the treatment effects up to 7% [25], and a meta-epidemiological study of blinding showed that unblinded RCTs overestimated the outcome effect by 0.56 standard deviations [26]. Blinding is helpful in eliminating intentional or unintentional bias, increasing the objectivity of results, and ensuring the credibility of study conclusions [24]. Although allocation concealment and blinding are critical research methodologies in RCTs, the 2012 compliance study of the CONSORT guidelines showed that only 12% and 21% of RCTs reported on allocation concealment and blinding in detail [27]. A study of acute herpes zoster showed that none of RCTs reported blinding and masking [28]. A study by Agha et al regarding the reporting quality of plastic surgery showed that the reporting rate for blinding was only 26% [29]. Therefore, it is urgent for researchers of randomized controlled trials to pay attention to the design and

implementation of allocation concealment and blinding , minimize intervention bias and measurement bias to improve the quality of RCTs. The estimation of sample size is related to the reliability, reproducibility and efficiency of the research results, which reflects the balance between clinical significance and statistics [30]. When the sample size calculation is adequately reported, the reader can understand what the study was designed to achieve [31]. In our study, only 5 studies (9.43%) reported the method of sample size estimation, it was similar to the findings in 40 RCTs of acute herpes zoster by Qian et al [28]. We suggest that researchers should attach great importance to the report of sample size estimation, so as to provide strong scientific evidence support for future clinical studies. Pre-planned subgroup analyses can be used to test intervention effects in groups. Only 7 studies reported subgroup analysis in our study [32]. In addition, due to the limited journal space, it is difficult to report the full details of trial process. Therefore, providing access to the trial protocol in the report of results is also an important measure to ensure the transparency of the trial.

Our study found that country of first author, types of interventions, journal of high impact factors and endorsement of the CONSORT statement were associated with reporting quality. We also found that the first author of the RCTs in China and America has the higher adherence score, which may be related to the outbreak of COVID-19 in China first. Due to the increasing number of confirmed and fatal cases, in order to control COVID-19 in a shorter period of time, China has paid more attention to this aspect than other countries. While articles from America were mostly published in journals with higher impact factor, such as *The New England journal of*

medicine, etc. For whatever reason, the reporting standards of RCTs regarding COVID-19 should not be ignored by researchers in any country. Some studies have shown that the number of author's centers is also associated with the reporting quality of RCTs [33,34], the articles we included were all multicenter studies. Studies from psychological intervention in China had the lower CONSORT cores and published in journals with impact factor less than 2. This is consistent with Shi's findings on the reporting quality of RCTs in Chinese nursing journals [35]. In our study, journal of higher impact factor and endorsement of the CONSORT statement were associated with better reporting quality. Results of Several previous studies also supported our findings [36-38]. In addition, a study in Germany showed that impact factors were also significantly correlated with the endorsement of the CONSORT statement [39]. This may be due to the fact that journals with higher impact factors have more stringent control about RCTs and higher recognition of the CONSORT statement. Thus, articles from journals which endorsed the CONSORT statement had greater reporting rate. This also means that if more journals are required to comply with CONSORT statement, the reporting of RCTs will improve. We suggest that researchers should attach great importance to the reporting standardization of RCT and strictly abide by the relevant provisions of the CONSORT statement when writing the paper. Editors should also strictly control the publication of RCTs, jointly improve the quality of RCTs, to provide scientific and strong evidence for clinical trials and meta-analysis. It is also hoped that more journals will endorse the CONSORT statement.

Conclusion

Our purpose is to provide the readers with a broad overview of the reporting characteristics of RCTs regarding patients or suspected patients with COVID-19. The overall reporting quality of RCTs regarding COVID-19 is suboptimal, thus diminishing their potential usefulness, and it is not conducive to the rapid and effective control of COVID-19. There remains room for improvement in the reporting quality of RCTs in COVID-19. The journal of high impact factors, endorsement of the CONSORT statement and China were associated with better reporting quality. More journals should accept the CONSORT statement, and journal reviewers should also strictly monitor the publication of RCTs. Future RCTs should particularly focus on improvement in allocation concealment, blinding and estimation of sample size.

Limitations of this study

There are some limitations in our study. First of all, this study only included relevant literature from 4 English database, it could not represent the overall reporting quality of RCTs regarding patients or suspected patient with COVID-19. In addition, although the literature retrieval, screening and quality evaluation were carried out simultaneously by two researchers, there was still some subjectivity.

Funding sources

This research did not receive any specific grant from funding agencies in the public, commercial, or not-for-profit sectors.

Authors contributions

The conception and design of the study: Yuhuan Yin.

Acquisition of data, analysis and interpretation of data: Yuhuan Yin, Juxia Zhang, Yiyin Zhang, Xiaoli Zhang, Jianying Ye.

Drafting the article: Yin Yuhuan.

Revising the article: Yuhuan Yin,Juxia Zhang.

Final approval of the article: Yuhuan Yin, Juxia Zhang, Yiyin Zhang, Xiaoli Zhang, Jianying Ye

Conflict of interest

No authors have any conflict of interest.

Acknowledgements

We thank Dr. Bin Ma (Evidence-Based Medicine Center, Institute of Traditional Chinese and Western Medicine, School of Basic Medical Sciences, Lanzhou University, Lanzhou, Gansu, China) for providing assistance with editing the final manuscript.

S1 Appendix. Search strategy

References

1. Sadeghi Dousari A, Taati Moghadam M, Satarzadeh N. COVID-19 (Coronavirus Disease 2019): A New Coronavirus Disease. Infection and drug resistance. 2020;13:2819-28. doi: 10.2147/IDR.S259279.
2. WHO. Coronavirus disease (COVID-2019) situation reports. 2020. https://www.who.int/emergencies/diseases/novel-coronavirus-2019/situation reports. [Cited 17 March 2020].
3. WHO. Coronavirus disease (COVID-2019). 2020. https://www.who.int/health-topics/coronavirus. [Cited 31 March 2020].
4. Guo YR, Cao QD, Hong ZS, Tan YY, Chen SD, Jin HJ, et al. The origin, transmission and clinical therapies on coronavirus disease 2019 (COVID-19) outbreak - an update on the status. Military Medical Research. 2020;7(1):11. doi: 10.1186/s40779-020-00240-0
5. Hajibandeh S, Hajibandeh S, Antoniou GA, Green PA, Maden M, Torella F. Reporting and Methodological Quality of Randomised Controlled Trials in Vascular and Endovascular Surgery. European journal of vascular and endovascular surgery : the official journal of the European Society for Vascular Surgery. 2015;50(5):664-70. doi:10.1016/j.ejvs.2015.06.114.

6. Hays M, Andrews M, Wilson R, Callender D, O'Malley PG, Douglas K. Reporting quality of randomised controlled trial abstracts among high-impact general medical journals: a review and analysis. BMJ open. 2016;6(7):e011082. doi: 10.1136/bmjopen-2016-011082.
7. Karpouzis F, Bonello R, Pribicevic M, Kalamir A, Brown BT. Quality of reporting of randomised controlled trials in chiropractic using the CONSORT checklist. Chiropractic & manual therapies. 2016;24:19. doi: 10.1186/s12998-016-0099-6.
8. Ntala C, Birmpili P, Worth A, Anderson NH, Sheikh A. The quality of reporting of randomised controlled trials in asthma: a systematic review. Primary care respiratory journal : journal of the General Practice Airways Group. 2013;22(4):417-24. doi: 10.4104/pcrj.2013.00089.
9. Begg C, Cho M, Eastwood S, Horton R, Moher D, Olkin I, et al. Improving the quality of reporting of randomized controlled trials. The CONSORT statement. Jama. 1996;276(8):637-9.
10. Moher D, Schulz KF, Altman D. The CONSORT statement: revised recommendations for improving the quality of reports of parallel-group randomized trials. Jama. 2001;285(15):1987-91. doi: 10.1001/jama.285.15.1987.
11. Schulz KF, Altman DG, Moher D. CONSORT 2010 statement: updated guidelines for reporting parallel group randomised trials. BMJ (Clinical research ed). 2010;340:c332. doi: 10.1136/bmj.c332 PMID:20332509.
12. CONSORT website. http://www.consort-statement/about-consort/endorses. [cited

18 March 2021].

13. Hajibandeh S, Hajibandeh S, Antoniou GA, Green PA, Maden M, Torella F. Association between bibliometric parameters, reporting and methodological quality of randomised controlled trials in vascular and endovascular surgery. Vascular. 2017;25(2):196-207. doi:10.1177/1708538116653289.
14. Huang YQ, Traore K, Ibrahim B, Sewitch MJ, Nguyen LHP. Reporting quality of randomized controlled trials in otolaryngology: review of adherence to the CONSORT statement. Journal of otolaryngology - head & neck surgery = Le Journal d'oto-rhino-laryngologie et de chirurgie cervico-faciale. 2018;47(1):34. doi: 10.1186/s40463-018-0277-8.
15. Yao AC, Khajuria A, Camm CF, Edison E, Agha R. The reporting quality of parallel randomised controlled trials in ophthalmic surgery in 2011: a systematic review. Eye (London, England). 2014;28(11):1341-9. doi:10.1038/eye.
16. Pratoomsoot C, Sruamsiri R, Dilokthornsakul P, Chaiyakunapruk N. Quality of reporting of randomised controlled trials of herbal interventions in ASEAN Plus Six Countries: a systematic review. PloS one. 2015;10(1):e108681. doi: 10.1371/journal.pone.0108681.
17. Akobeng AK. Understanding randomised controlled trials. Archives of disease in childhood. 2005;90(8):840-4. doi: 10.1136/adc.2004.058222.
18. Chow DS, Ha R, Filippi CG. Increased rates of authorship in radiology publications: a bibliometric analysis of 142,576 articles published worldwide by radiologists between 1991 and 2012. AJR American journal of roentgenology.

2015;204(1):W52-7. doi: 10.2214/AJR.14.12852.

19. Sterne JA, Egger M. Funnel plots for detecting bias in meta-analysis: guidelines on choice of axis. Journal of clinical epidemiology. 2001;54(10):1046-55. doi: 10.1016/s0895-4356(01)00377-8.
20. Chan AW, Hróbjartsson A, Haahr MT, Gøtzsche PC, Altman DG. Empirical evidence for selective reporting of outcomes in randomized trials: comparison of protocols to published articles. Jama. 2004;291(20):2457-65.
21. Dechartres A, Trinquart L, Boutron I, Ravaud P. Influence of trial sample size on treatment effect estimates: meta-epidemiological study. BMJ (Clinical research ed). 2013;346:f2304. doi: 10.1136/bmj.f2304.
22. Bhide A, Shah PS, Acharya G. A simplified guide to randomized controlled trials. Acta obstetricia et gynecologica Scandinavica. 2018;97(4):380-7. doi: 10.1111/aogs.13309.
23. Calvert M, Blazeby J, Altman DG, Revicki DA, Moher D, Brundage MD. Reporting of patient-reported outcomes in randomized trials: the CONSORT PRO extension. Jama. 2013;309(8):814-22. doi: 10.1001/jama.2013.879.
24. Sil A, Kumar P, Kumar R, Das NK. Selection of Control, Randomization, Blinding, and Allocation Concealment. Indian dermatology online journal. 2019;10(5):601-5. doi: 10.4103/idoj.IDOJ_149_19.
25. Savović J, Jones H, Altman D, Harris R, Jűni P, Pildal J, et al. Influence of reported study design characteristics on intervention effect estimates from randomised controlled trials: combined analysis of meta-epidemiological studies.

Health technology assessment (Winchester, England). 2012;16(35):1-82. doi: 10.3310/hta16350.

26. Hróbjartsson A, Emanuelsson F, Skou Thomsen AS, Hilden J, Brorson S. Bias due to lack of patient blinding in clinical trials. A systematic review of trials randomizing patients to blind and nonblind sub-studies. International journal of epidemiology. 2014;43(4):1272-83. doi: 10.1093/ije/dyu115.

27. Ghimire S, Kyung E, Kang W, Kim E. Assessment of adherence to the CONSORT statement for quality of reports on randomized controlled trial abstracts from four high-impact general medical journals. Trials. 2012;13:77. doi: 10.1186/1745-6215-13-77.

28. Qian G, Zeng J, Lu L, Pei W, Liu K, Luo Z, et al. Evaluation of Reporting Quality in Randomised Controlled Trials of Acupuncture for Acute Herpes Zoster by the CONSORT Statement and STRICTA Guidelines. Evidence-based complementary and alternative medicine : eCAM. 2020;2020:4308380. doi: 10.1155/2020/4308380.

29. Agha RA, Camm CF, Doganay E, Edison E, Siddiqui MR, Orgill DP. Randomised controlled trials in plastic surgery: a systematic review of reporting quality. European journal of plastic surgery. 2014;37(2):55-62. doi: 10.1007/s00238-013-0893-5.

30. Weber EJ, Hoo ZH. Why sample size estimates? Emergency medicine journal : EMJ. 2018;35(12):755-6. doi: 10.1136/emermed-2018-207763.

31. Copsey B, Thompson JY, Vadher K, Ali U, Dutton SJ, Fitzpatrick R, et al. Sample

size calculations are poorly conducted and reported in many randomized trials of hip and knee osteoarthritis: results of a systematic review. Journal of clinical epidemiology. 2018;104:52-61. doi: 10.1016/j.jclinepi.2018.08.013.

32. Petkovic J, Jull J, Yoganathan M, Dewidar O, Baird S, Grimshaw JM, et al. Reporting of health equity considerations in cluster and individually randomized trials. Trials. 2020;21(1):308. doi: 10.1186/s13063-020-4223-5.
33. Chen W, Yu J, Zhang L, Su G, Wang W, Kwong J, et al. Quality of reporting in randomized controlled trials of therapeutic cardiovascular medical devices. Surgery. 2019;165(5):965-9. doi: 10.1016/j.surg.2018.09.010.
34. Chen J, Li Z, Liu B, Gan X, Li C, Yu H. Quality improvement in randomized controlled trial abstracts in prosthodontics since the publication of CONSORT guideline for abstracts: a systematic review. Journal of dentistry. 2018:23-9. doi: 10.1016/j.jdent.2018.04.025.
35. Shi C, Tian J, Ren D, Wei H, Zhang L, Wang Q, et al. Methodological reporting of randomized trials in five leading Chinese nursing journals. PloS one. 2014;9(11):e113002. doi: 10.1371/journal.pone.0113002.
36. Canagarajah NA, Porter GJ, Mitra K, Chu TSM. Reporting quality of surgical randomised controlled trials in head and neck cancer: a systematic review. European archives of oto-rhino-laryngology : official journal of the European Federation of Oto-Rhino-Laryngological Societies (EUFOS) : affiliated with the German Society for Oto-Rhino-Laryngology - Head and Neck Surgery. 2021. doi: 10.1007/s00405-021-06694-9.

37. Bigna JJ, Noubiap JJ, Asangbeh SL, Um LN, Sime PS, Temfack E, et al. Abstracts reporting of HIV/AIDS randomized controlled trials in general medicine and infectious diseases journals: completeness to date and improvement in the quality since CONSORT extension for abstracts. BMC medical research methodology. 2016;16(1):138. doi: 10.1186/s12874-016-0243-y.

38. Vassar M, Jellison S, Wendelbo H, Wayant C, Gray H, Bibens M. Using the CONSORT statement to evaluate the completeness of reporting of addiction randomised trials: a cross-sectional review. BMJ open. 2019;9(9):e032024. doi: 10.1136/bmjopen-2019-032024.

39. Stevanovic A, Schmitz S, Rossaint R, Schürholz T, Coburn M. CONSORT item reporting quality in the top ten ranked journals of critical care medicine in 2011: a retrospective analysis. PloS one. 2015;10(5):e0128061. doi:10.1371/journal.pone.0128061.

Click here to access/download
Supporting Information
S1 Appendix.docx
