# Supplementary material for: Evaluation of reporting quality of randomized controlled trials in patients with COVID-19 using the CONSORT statement
Source: PLoS One. 2021 Sep 23;16(9):e0257093. doi: 10.1371/journal.pone.0257093 (PMC8460279; doi:10.1371/journal.pone.0257093)
Supplement: Attachment — Submitted filename: Response to Reviewers.docx [file pone.0257093.s003.docx]

Dear reviewer

We appreciate the positive comments which you made. Our responses to the comments are as follows:

**Reviewer #1**

While the data appears to be well done and I feel the topic is germaine, there are numerous issues with noun-verb agreement, there are a few cases where two sentences should be one, as well as multiple other grammatical issues. I highlighted a few of these in the attached document. However, they were too numerous to highlight them all.

**Answer:** We have reconstructed the grammar and writing in accordance with reviewer, please check.

**Reviewer #2**

1. There is no mention of inter-rater reliability when judging the items. This is a major and recurring problem in studies that attempt to measure whether trials have adhered to reporting guidelines or not. There is a lot of subjective judgement involved in making a decision. For this to be robust and rigorous, the assessors should be trained and standardized.

**Answer:** We have calculated inter-rater reliability in accordance with reviewer, the result was showed in page 8, please check.

2. For instance, some of the items are marked as 0% adherence to CONSORT. One of the items is with regards to participant exclusion and losses after randomization. I can't believe this can be true, because any trial that has the CONSORT flowchart will definitely have reported this information. Also, there are a couple of CONSORT items which ask to report any changes to methods or outcomes, if any. These were marked as 0%. However, if they trialists had no changes, they would not have reported it, so it seems to me somewhat inaccurate to mark these as 0%.

**Answer:** Because we initially had a wrong understanding of the item of participant exclusion and losses after randomization, we focused on the description of the results and ignored the information in the CONSORT flow chart. According to the reviewer’s opinion, we extracted the information of this item again (Table 2), and the results are shown in Table 2, please check.

Other items reported as 0% are described in the methods section on page 6, items 3b, 6b, and 14b are not necessarily applicable, if relevant, the article will be graded according to the above guidelines, if the article did not apply to this item, no points was deducted, please check.

3. A lot of the summary descriptive statistics report 'average' scores etc. I don't necessarily think the mean or average is a suitable summary statistic here given the likelihood of markedly skewed data.

**Answer:** We have removed the summary statistical report of ‘average’ scores in accordance with reviewer, please check.

4. The discussion is much too long. I don't get a feel why this topic is important in context of other studies that have already demonstrated variable levels of adherence to CONSORT. I don't see that trials in COVID are any different from trials in cancer, surgery, diabetes, heart disease etc. I think the analysis is unnecessarily complicated and there are too many variables being evaluated in a fishing expedition. I don't see that number of authors etc. has any bearing on CONSORT adherence for instance, and I can't understand why it was even entered into the statistical analysis.

**Answer:** As no study has specifically evaluated the reporting quality of RCTs regarding patients with COVID-19. The primary objective of our study was to assess the reporting quality of RCTs regarding patients with COVID-19 and analyze possible related factors, so as to provide more references for high-quality research. We have pruned the discussion part according to the reviewer's opinion, please check.

According to the comments of reviewers, we readjusted the variable analysis. We conducted univariate analysis in Table 1, factors that were statistically significant in univariate analysis were included in multiple regression models, the result was showed Table 3 (page 12), please check.
